# The Effect of Glycemic Control on Morbidity and Mortality in Critically Ill COVID-19 Patients

**DOI:** 10.7759/cureus.47991

**Published:** 2023-10-30

**Authors:** Kinza Sultan, Sarala Kal, Leo Issagholian, Birpartap S Thind, Sarah C Neeki, Hovhannes Ghazaryan, Alex Jabourian, Fanglong Dong, Ho-Wang Yuen, Sarkis Arabian, Michael Neeki

**Affiliations:** 1 Internal Medicine, Arrowhead Regional Medical Center, Colton, USA; 2 Medicine, California University of Science and Medicine, Colton, USA; 3 Emergency Medicine, Arrowhead Regional Medical Center, Colton, USA; 4 Clinical Research, Western University of Health Sciences, Pomona, USA; 5 Biomedical Sciences, Western University of Health Sciences, Pomona, USA; 6 Emergency Medicine, California University of Science and Medicine, Colton, USA

**Keywords:** glycemic index, angiotensin converting enzyme, intensive care unit, diabetes mellitus, covid-19

## Abstract

Background

COVID-19 infection has caused a global pandemic affecting a group of patients with chronic conditions including diabetes with exacerbating insulin resistance and hyperglycemia. Investigators noted that pre-existing diabetes and newly diagnosed diabetes are associated with an increased risk of all-cause mortality in hospitalized patients with COVID-19 infection.

Aim

To evaluate the relationship between ICU patients infected with COVID-19 and mortality among those with high versus low glucose levels.

Methods

This is a retrospective study of critically ill adult patients infected with COVID-19 who were admitted to the ICU from April 5, 2020, to October 14, 2020. The participants were from San Bernardino County which is a diverse and underserved community. Overall, 84 patients were included in the final analysis. The average age was 59.67 (standard deviation=15.55) with 59.5% being males. Overall mortality was 44.1%.

Results

Around one-fifth of patients had glucose under control as measured by peak glucose level of <180 mg/dL during hospital stay. A statistically significant association was seen between tighter serum glucose control and mortality (p=0.0354). Patients with serum glucose maintained <180 mg/dL were associated with significantly lower mortality than their counterparts (22.2% vs. 50%).

Conclusions

This study suggests that maintaining a tighter control of the glycemic index in critically ill COVID-19 patients will improve morbidity and mortality.

## Introduction

COVID-19 is caused by a single-stranded RNA virus called SARS-CoV-2 [1]. COVID-19 was declared a global pandemic in March of 2020, and since then, WHO has recognized 768 million cases with almost seven million deaths worldwide, with 100 million cases in the United States (US) alone [2]. COVID-19 presentation is characterized by cold symptoms involving fever, cough, dyspnea, and occasional gastrointestinal symptoms [3]. This vague and generalized symptomology leads to broad differentials, and the diagnosis is usually clinical, though, with the vast availability of testing kits, many patients can be quickly and accurately tested [4]. The mortality rate of COVID-19 has been variable in the existing literature.

Early courses of COVID-19 were especially deadly due to the novelty of the virus and lack of established treatment protocols and vaccinations; however, certain populations including the elderly, diabetes mellitus (DM), and ICU patients showed a higher likelihood of worse outcomes [3]. Bode et al. noted an inherent correlation between glycemic variation among diabetic patients hospitalized due to COVID-19 infection [5]. Furthermore, they noted that diabetic patients admitted to hospitals for COVID-19 had higher sustained serum glucose levels (200 vs. 115 mg/dL) and worse renal function than those without a history of DM [5]. Investigators also suggested that the poor outcomes in diabetic patients are likely a result of them effectively being in an immunocompromised state [6]. Furthermore, diabetic patients infected with COVID-19 have a higher rate of admission to the ICU [5]. Studies also reported a higher rate of deep venous thromboembolism with the occurrence of pulmonary embolism [7] and a higher odds ratio of hospital mortality and cardiovascular injury [1].

Of note, a large meta-analysis reported that a history of COVID-19 infection exposed the patients to a 1.8 relative risk for new onset of type 2 DM with the greatest risk being within three months of infection [8]. The authors suggested this could be related to systemic inflammation and insulin resistance, though a true molecular mechanism has yet to be elucidated. Similarly, a large retrospective review also noted that patients infected with COVID-19 had a 1.8 OR of developing DM within 90 days [9]. This OR was not observed in vaccinated individuals. Various theories were suggested to explain these observations, which included direct viral infection and destruction of islet cells, to an autoimmune activation by the cytokine storm in severe COVID-19 encounters; however, no single mechanism has been validated thus far [8,10].

Previous literature provided a large body of evidence regarding the relationship between COVID-19 and DM. Some studies evaluated the mortality rate in the ICU [10]. Those patients who were admitted to the ICU and maintained consistently high blood glucose were found to have worse outcomes [5]. This retrospective aims to evaluate the outcomes in patients admitted to the ICU at a large safety net regional hospital for COVID-19 at the height of the pandemic.

## Materials and methods

This is a retrospective study of critically ill adult patients infected with COVID-19 admitted directly from the emergency department to the ICU. Patients were seen at Arrowhead Regional Medical Center (ARMC) from April 5, 2020, to October 14, 2020. ARMC is a 456-bed university-affiliated teaching public hospital that serves as a safety net hospital for the uninsured and underserved population in San Bernardino County, California. ARMC has multiple sub-specialty ICUs. The medical ICU had approximately 3000 patients per year. Per the US census, San Bernardino County is the largest geographic county in the continental United States, with a diverse population of 2.2 million people.

A total of 514 patients were admitted to the ICU with SARS-CoV-2 infection during the given time range. Patients were included in the study if they tested positive for SARS-CoV-2 on a real-time polymerase chain reaction test (RT-PCR) upon admission to the hospital ICU. Patients were included in the study if they were sustained on endotracheal intubation with mechanical ventilation or non-invasive mechanical ventilation when directly admitted to the ICU. Of these patients admitted to the ICU, 84 patients met the inclusion criteria. Patients were excluded if they did not meet the above criteria, if they had another primary diagnosis, or if otherwise transferred for the care of another concomitant etiology. The investigating team members performed a detailed review of each patient's electronic medical chart to identify participants who matched the study's defined criteria. Two researchers performed an analysis of patient charts to ensure adequate data extraction. Special care was taken to remove identifiable health data. This study was reviewed and approved by the Institutional Review Board of Arrowhead Regional Medical Center with the approval number IRB# 22-22.

Two groups were created for comparison purposes. The first group was for patients with peak glucose level <180 milligrams per deciliter (mg/DL) and the second group was for patients with peak glucose >=180 mg/DL. The primary outcome was mortality, which is defined as if patients expired at any point during the study's duration or while in the ICU stay. Other demographic variables (e.g., age and gender) and clinical variables (e.g., HbA1c) were also compared with the two peak glucose groups.

All statistical analyses were conducted using the SAS software for Windows version 9.4 (Cary, North Carolina, USA). Descriptive statistics were presented as means and standard deviations for continuous variables, along with frequencies and proportions for categorical variables. Chi-square crosstab analyses were conducted to assess the association between categorical variables and the binary glycemic control groups. Independent t-tests were conducted to assess the difference in continuous variables between the binary glycemic control groups. All statistical analyses were two-sided. A p-value of <0.05 was statistically significant.

## Results

A total of 84 patients were included in the final analysis. The average age was 59.67 (SD=15.55) years with 59.5% (n=50) of patients being males. The average BMI was 31.98 (SD=9.07). The overall mortality was 44.1%. Nearly one-fifth (21.4%) had their glucose under control as measured by the peak glucose level <180 mg/dL during their hospital stay. Table 1 presents the demographic summary.

**Table 1:** Demographic summary and comparison of variables HbA1c: hemoglobin A1c, BMI: body mass index, APACHE: acute physiology and chronic health evaluation, SOFA: Sequential Organ Failure Assessment, P significance is less than 0.05

		Peak glucose level below 180 (n=18)	Peak glucose level above 180 (n=66)	p-value
Mortality				0.0354
Alive	47 (56%)	14 (77.8%)	33 (50%)	
Dead	37 (44.1%)	4 (22.2%)	33 (50%)	
Gender				0.1414
Female	34 (40.5%)	10 (55.6%)	24 (36.4%)	
Male	50 (59.5%)	8 (44.4%)	42 (63.6%)	
HbA1c				0.0009
HbA1c below 6.5	26 (35.1%)	9 (81.8%)	17 (27%)	
HbA1c above 6.5	48 (64.9%)	2 (18.2%)	46 (73%)	
Procalcitonin				0.3265
Procalcitonin below 2	59 (90.8%)	14 (100%)	45 (88.2%)	
Procalcitonin above 2	6 (9.2%)	0 (0%)	6 (11.8%)	
Age	59.67 ± 15.55	56.28 ± 18.49	60.59 ± 14.67	0.2996
BMI	31.98 ± 9.07	33.85 ± 8.81	31.45 ± 9.14	0.3385
APACHE score	1.55 ± 6.89	3.83 ± 13.07	0.93 ± 3.74	0.1136
SOFA score	4.02 ± 2.38	4.22 ± 2.67	3.97 ± 2.31	0.6923

There was a statistically significant association between glucose control and mortality (p=0.0354). Specifically, patients with glucose controlled under the current recommended ICU guidelines were associated with significantly lower mortality as compared to their counterparts (22.2% vs. 50%). Intuitively, patients who have a peak glucose level <180 were associated with a lower percentage of HbA1c ≤6.5. No other statistically significant differences were identified in the analysis. See Table 1 for the detailed analysis results.

## Discussion

The finding in this study aligns with the existing literature, highlighting that COVID-19 patients with pre-existing diabetes are at a higher risk of severe illness, hospitalization, and mortality compared to those without diabetes [8,11]. The exacerbation of hyperglycemia in infected COVID-19 diabetic patients may be explained by a proposed mechanism involving the entry of SARS-CoV-2 through ACE2 receptors in pancreatic beta islet cells [5]. Macedo et al. reported that the majority of ACE2 receptor expression is found in the lungs, consistent with the clinical picture of COVID-19 including acute respiratory distress syndrome; however, off-target effects are likely secondary to the above mechanism [12]. It should be noted, however, that there have been some concerns about the potential impact on glycemic control and the development of type 1 DM from off-target effects as the evidence is not entirely clear and at times conflicting [10,13]. Therefore, a definitive association between COVID-19 and the development of type 1 DM has not been clearly established [11].

Furthermore, published literature suggests that diabetes may not only be a risk factor for COVID-19 but also a condition that may exacerbate during the disease process [14]. Investigators also noted a possible association between elevated blood glucose levels and increased viral load, underscoring the significance of glycemic control in managing COVID-19 in patients with diabetes [8,10,15]. As a result, serum glycemic control is thought to be an important factor in improving the outcome in critically ill COVID-19 patients with pre-existing diabetes [8,11]. In line with this idea, this study also revealed a significant association between glycemic control and mortality in critically ill COVID-19 patients with pre-existing diabetes. Patients with a peak glucose level <180 mg/dL during their hospital stay experienced markedly lower mortality rates than those with uncontrolled glucose levels, highlighting the critical role of maintaining appropriate glycemic control in this patient population [8,11].

It is important to note that along with uncontrolled serum glucose, other variables such as disease severity and age may also contribute to increased mortality rates in critically ill COVID-19 patients with pre-existing diabetes [16]. While glycemic control and its contribution to mortality were noted, it is also essential to consider the correlation of the Sequential Organ Failure Assessment (SOFA) Score in ICU patients. SOFA scores can help assess a patient's risk, provide valuable information for predicting outcomes in critically ill patients, and allow the identification of patients at higher risks [17]. In line with findings from Assal et al., the results of this study revealed a significant association between a higher SOFA score and mortality in patients with profound disseminated disease [16]. This may strengthen the idea that the disease severity and altered immune system with inflammatory status may play a key role in critically ill COVID-19 patients with pre-existing diabetes [13]. Moreover, understanding how SARS-CoV-2 alters immunity and inflammatory status adds to our understanding of the disease's progression in critically ill patients with pre-existing diabetes [13]. The results of this study did not substantiate such a relationship; however, this may be due to the low number of study subjects.

It is essential to acknowledge several limitations in this study that merit discussion. First, the retrospective design may have introduced selection bias and limited the availability of comprehensive data, potentially affecting the generalizability of the results. Additionally, the sample size could have impacted the study's power to detect subtle associations. Moreover, external factors, such as comorbidities, variability in the treatment protocols, and individual responses to the virus, may have influenced the outcomes and should be considered in future investigations. Nonetheless, this study contributes to the growing body of knowledge on the importance of glycemic control in critically ill COVID-19 patients with pre-existing diabetes.

## Conclusions

This study noted that glycemic control may be an important factor in determining the outcome of critically ill COVID-19 patients with pre-existing diabetes. These findings highlight the importance of following more reasonable glycemic control regimens based on the current recommendations for improving outcomes in COVID-19 patients with pre-existing diabetes. Further research is needed to evaluate the impact of different glycemic control strategies on mortality in COVID-19 patients.
